# Chronic Ureteral Compression From a Thrombosed Iliac Artery Aneurysm: A Manifestation of Aortoiliac Aneurysmal Disease

**DOI:** 10.7759/cureus.99963

**Published:** 2025-12-23

**Authors:** Ahmad Jalil, Venkata Varshitha Bandi, Dhairya Shah, Vikram Beemidi

**Affiliations:** 1 Internal Medicine, Baptist Memorial Hospital, North Mississippi, Oxford, USA; 2 Nephrology, Baptist Memorial Hospital, North Mississippi, Oxford, USA

**Keywords:** aortoiliac aneurysmal disease, extrinsic ureteral compression, hydronephrosis, iliac artery aneurysm, ureteral obstruction

## Abstract

Ureteral obstruction caused by iliac artery aneurysms is uncommon and typically results from acute aneurysmal expansion or rupture. We describe a rare presentation in which chronic external compression from a large, thrombosed iliac artery aneurysm led to persistent left-sided hydronephrosis requiring serial ureteral stent exchanges in a 69-year-old male patient. The patient had an extensive vascular history, including prior aortoiliac aneurysm repair, and was not a candidate for further surgical intervention. Sequential CT imaging consistently demonstrated a stable, multilobulated thrombosed internal iliac artery aneurysm displacing the left ureter into a tortuous course. This case highlights an unusual mechanism of chronic ureteral obstruction and underscores the importance of recognizing vascular causes in patients with recurrent obstruction and complex vascular anatomy.

## Introduction

Iliac artery aneurysms are uncommon vascular lesions that can present with urologic complications when their proximity to the ureter results in external compression and hydronephrosis [1]. Although often asymptomatic, these aneurysms may cause flank pain, renal colic, or obstructive uropathy, particularly when large or thrombosed [2]. Ureteral obstruction from iliac artery aneurysms has been documented in both solitary and reconstructed aortoiliac systems [3,4]. Depending on size and anatomy, they may produce ipsilateral hydronephrosis, ureteral deviation, or mass effect on adjacent pelvic structures [5]. Early recognition is essential to prevent progressive renal compromise and guide appropriate multidisciplinary management. Isolated iliac artery aneurysms are uncommon, accounting for roughly 2% of all aortoiliac aneurysms and approximately 5.0% of cases reported in surgical series [6,7]. In contrast, common iliac artery aneurysms associated with abdominal aortic aneurysm (AAA) are considerably more frequent, with surveillance studies indicating a prevalence of 20%-40% among patients with AAA [8]. However, chronic ureteral obstruction caused by long-standing external compression from a stable thrombosed iliac artery aneurysm, particularly in patients who are not candidates for definitive vascular repair, remains poorly characterized in the existing literature.

## Case presentation

A 69-year-old man with a medical history of end-stage renal disease on hemodialysis, abdominal aortic aneurysm with prior vascular grafting including previous aortoiliac aneurysm repair, coronary artery disease status post coronary artery bypass grafting, stroke with residual deficits, and chronic left ureteral obstruction presented with left flank pain. Initial CT imaging performed at an outside facility demonstrated left-sided hydronephrosis with abrupt proximal ureteral obstruction without an identifiable stone. A Foley catheter yielded minimal urine output despite his baseline ability to produce small amounts of urine.

He was transferred to our hospital, where cystoscopy, retrograde pyelogram, and the procedure of placement of a left ureteral stent were performed by Urology. Urology expressed concern for external ureteral compression, possibly related to his complex vascular anatomy, and recommended ongoing surveillance. A CT angiogram obtained shortly after transfer showed tortuous, ectatic aortoiliac vasculature and a lobulated left pelvic mass abutting the iliac vessels, initially labeled indeterminate. The left nephroureteral stent was visualized with persistent dilatation of the left renal pelvis, and no stone or intrinsic ureteral lesion was identified.

**Figure 1:**
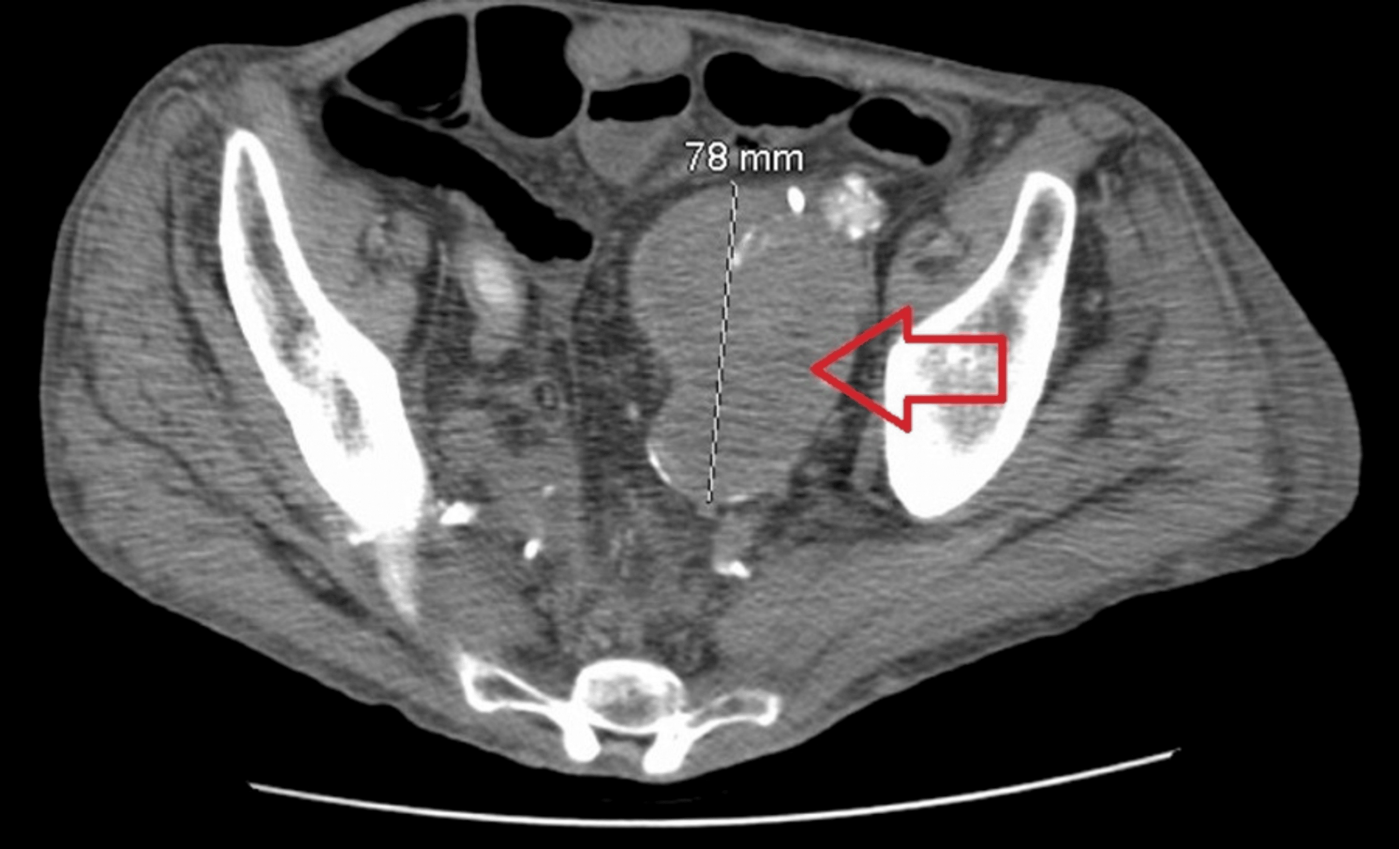
The mass in the left hemipelvis (red arrow) representing a large, thrombosed aneurysm, involving the internal iliac artery. No blood flow was seen in the mass.

**Figure 2:**
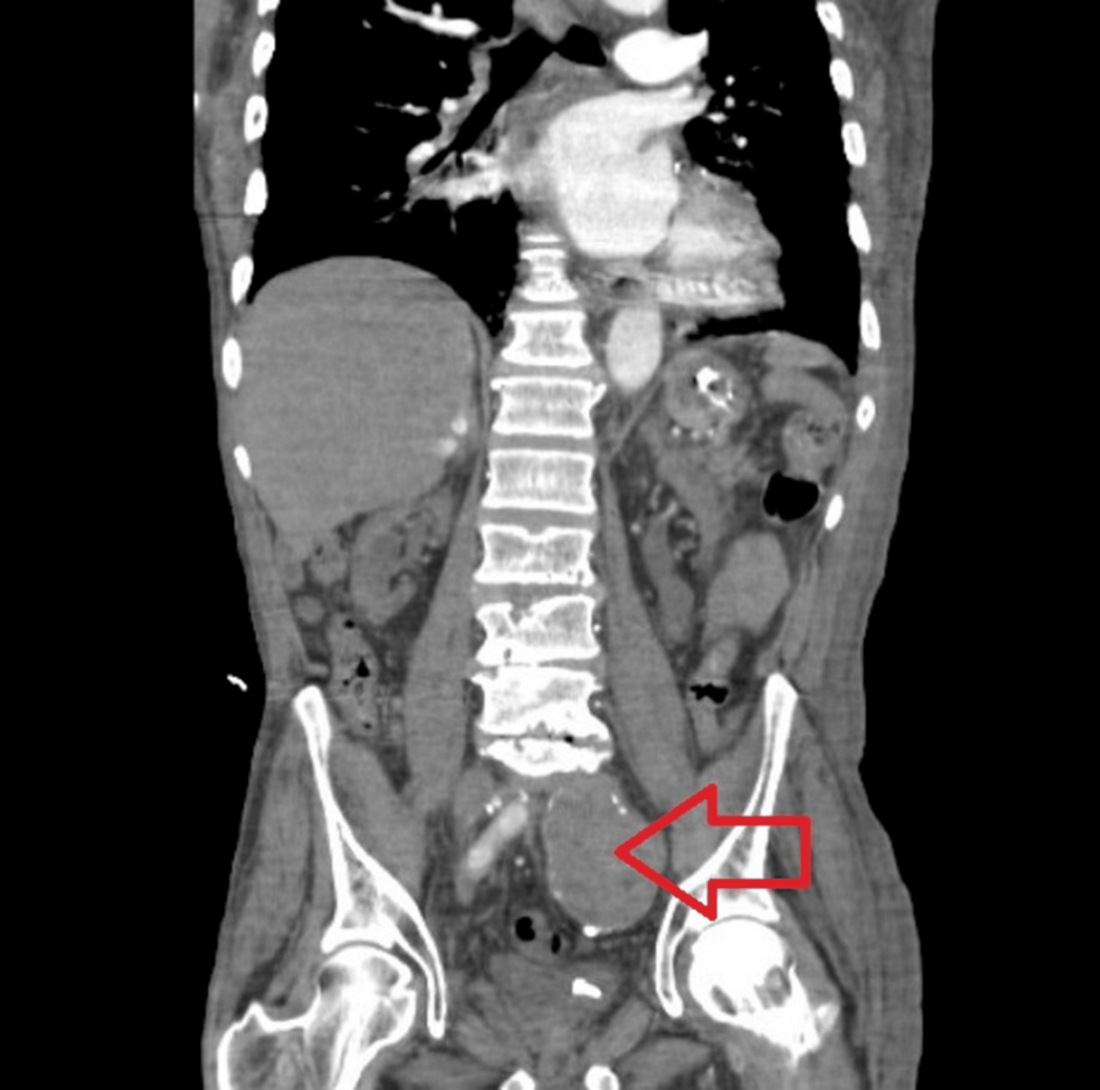
Large thrombosed aneurysm of the iliac artery on the left side, shown by a red arrow

Over the following year, the patient experienced multiple hospitalizations for recurrent abdominal symptoms, including nausea, vomiting, and suspected small bowel obstruction. Serial CT imaging consistently demonstrated a large, multilobulated mass in the left hemipelvis measuring approximately 7.6 to 7.8 cm, with no interval change in size, and showing peripheral calcification and mass effect on adjacent structures. Subsequent imaging definitively characterized this lesion as a large, thrombosed aneurysm of the left internal iliac artery with extension into the distal common iliac artery, as shown in Figures 1-2.

The aneurysm caused significant anatomic distortion, displacing the left ureter into a markedly tortuous course around the thrombosed sac. Given the close anatomic relationship between the iliac vessels and the ureter in the pelvis, the thrombosed aneurysm exerted sustained extrinsic compression. A left renal artery stent and prior aorto-femoral and aorto-iliac bypass grafts were also noted.

Despite remaining stable in size, the aneurysm continued to exert external compression on the left ureter, resulting in persistent hydronephrosis that required periodic ureteral stent exchanges approximately every eight months. Although the patient remained dialysis dependent, he continued to produce limited urine output, making urinary decompression clinically important. Multidisciplinary evaluation by Urology and Vascular Surgery determined that he was a poor candidate for definitive aneurysm repair due to extensive comorbidities and prior vascular reconstruction.

This case illustrates a rare presentation in which a chronically thrombosed, nonexpanding iliac artery aneurysm created persistent mechanical ureteral compression rather than the more commonly reported mechanism of acute aneurysmal expansion or rupture. It further highlights that in patients with prohibitive surgical risk from comorbidities, including those related to prior reconstruction, long-term palliative ureteral stenting may represent the most feasible management strategy. The patient continues to receive periodic stent exchanges with multidisciplinary follow-up.

## Discussion

Iliac artery aneurysms are rare vascular lesions, but their proximity to the ureter can result in ureteral obstruction and subsequent hydronephrosis [1]. Clinical presentation often includes flank pain, renal colic, or nonspecific abdominal symptoms, and may be complicated by hematuria or acute kidney injury if obstruction is severe [2,3]. In some cases, a palpable pulsatile mass or bruit may be detected on physical examination [4].

The pathophysiology involves either direct mechanical compression of the ureter by the expanding aneurysm or, less commonly, secondary retroperitoneal fibrosis induced by inflammatory changes surrounding the aneurysm [4,5]. This extrinsic compression leads to impaired urinary drainage, resulting in hydronephrosis, which can be unilateral or, in rare cases, bilateral if the aneurysm or associated hematoma is large enough to affect both ureters [4,5,9]. Imaging modalities such as CT, MRI, and intravenous pyelography are essential for diagnosis, revealing the aneurysm and its relationship with the ureter, as well as the degree of hydronephrosis [5,10,11].

Management strategies must address both the vascular and urologic complications. Historically, open surgical repair was the mainstay, often combined with ureterolysis to free the ureter from surrounding tissue and, if necessary, temporary urinary diversion to protect renal function [4,11,12]. More recently, endovascular aneurysm repair (EVAR) has emerged as a less invasive alternative, with case series demonstrating successful resolution of urinary obstruction and reduction in aneurysm size [1]. Although EVAR offers a minimally invasive option and has demonstrated good early outcomes in non-inflammatory iliac artery aneurysms associated with urinary obstruction, its long-term effectiveness is not yet well established. In cases where the aneurysm is accompanied by marked inflammation or extensive retroperitoneal fibrosis, open surgical repair with ureterolysis is often favored because it allows surgeons to excise the fibrotic tissue directly and provides a more definitive and durable resolution of ureteral compression [1,11,13,14].

Urologic intervention may be required for acute obstruction or renal compromise. Placement of a ureteral stent or percutaneous nephrostomy can provide temporary relief and preserve renal function until definitive vascular repair is performed [12]. In rare cases, ureteral transposition or reconstruction may be necessary if the ureter is extensively involved or damaged [15].

Prognosis depends on timely recognition and intervention. Delayed treatment can result in permanent nephron loss or life-threatening complications such as aneurysm rupture, which carries a high mortality rate [16]. Clinicians should maintain a high index of suspicion for iliac artery aneurysms in patients presenting with unexplained hydronephrosis and a pelvic mass, as atypical or chronic aneurysms can cause persistent ureteral compression even in the absence of acute expansion [16,17].

## Conclusions

Chronic ureteral obstruction secondary to a thrombosed iliac artery aneurysm is rare and easily overlooked, as symptoms may mimic primary urologic disease. This case underscores the importance of maintaining a broad differential diagnosis when evaluating hydronephrosis, especially in patients with prior vascular reconstruction. Recognition of underlying vascular compression allows targeted management and appropriate surveillance. Importantly, this case highlights that a chronic, stable, nonexpanding iliac artery aneurysm can produce persistent ureteral compression, and in patients who are not surgical candidates, periodic ureteral stent exchanges may be required to preserve residual renal function and maintain symptomatic relief.
